# Postoperative Sciatic Neuropathy Following Spinal Anesthesia in a Patient With Severe Lumbar Degenerative Disease: A Case Report

**DOI:** 10.7759/cureus.113046

**Published:** 2026-07-20

**Authors:** Woojin Nam, Kyeongyoon Woo

**Affiliations:** 1 Anesthesiology and Pain Medicine, Daegu Fatima Hospital, Daegu, KOR; 2 Anesthesiology, Daegu Fatima Hospital, Daegu, KOR

**Keywords:** electrodiagnostic study, lumbar spinal stenosis, peripheral nerve injury, postoperative neurologic deficit, sciatic neuropathy, spinal anesthesia, spondylolisthesis, tibial division neuropathy

## Abstract

Neurologic complications after spinal anesthesia are uncommon but remain a major concern because of their potential severity. Distinguishing anesthesia-related neurologic injury from preexisting spinal pathology is often challenging.

A 70-year-old man with hypertension, hyperlipidemia, and known lumbar degenerative disease underwent elective bilateral inguinal hernia repair under spinal anesthesia. Spinal anesthesia was performed via a paramedian approach at the L3-4 interspace using a 25-gauge Quincke needle and 2.6 mL (13 mg) of 0.5% hyperbaric bupivacaine without paresthesia or technical difficulty. Sensory block reached the T6 dermatome level. The intraoperative course was uneventful, with stable hemodynamics throughout the procedure.

Approximately two hours after surgery, motor function had recovered in all extremities except the right lower limb. The neurologic deficits persisted despite completion of the standard six-hour postoperative bed rest, with continued sensory loss, paresthesia, and motor weakness in the right lower extremity, whereas the left lower extremity recovered completely. Lumbar magnetic resonance imaging demonstrated severe degenerative lumbar disease, including marked L5-S1 spondylolisthesis, epidural fat hypertrophy, and central canal narrowing, without evidence of neuraxial hematoma, abscess, or other compressive lesions. Serial neurologic examinations demonstrated gradual improvement. Electrodiagnostic studies subsequently revealed findings consistent with right sciatic neuropathy with predominant tibial division involvement.

Although a causal relationship between spinal anesthesia and postoperative neuropathy could not be definitively established, perioperative positioning-related sciatic neuropathy was also considered in the differential diagnosis but was regarded as less likely based on the operative course.

## Introduction

Spinal anesthesia is one of the most commonly performed regional anesthetic techniques and is generally considered safe and effective for lower abdominal, urologic, orthopedic, and lower extremity procedures [1,2]. Serious neurologic complications are uncommon, but when they occur, they may result in significant morbidity and raise concerns regarding anesthesia-related nerve injury [1,3].

Postoperative neurologic deficits following spinal anesthesia may arise from a variety of etiologies, including direct needle trauma, neurotoxicity of local anesthetics, epidural hematoma, epidural abscess, ischemic injury, preexisting neurologic disease, or peripheral nerve injury unrelated to neuraxial anesthesia, including perioperative positioning- or compression-related neuropathy [1,3,4]. Because the temporal relationship between spinal anesthesia and symptom onset is often striking, distinguishing causation from coincidence can be challenging in clinical practice [2,4].

Patients with underlying spinal pathology, such as lumbar spinal stenosis, degenerative spondylosis, epidural lipomatosis, or spondylolisthesis, may be particularly vulnerable to postoperative neurologic symptoms [1,2]. Previous studies have suggested that preexisting narrowing of the spinal canal may reduce neural reserve and increase susceptibility to neurologic dysfunction following neuraxial procedures [1]. However, establishing a direct causal relationship remains difficult, especially when imaging studies fail to identify an acute neuraxial lesion [2,3].

Sciatic neuropathy is an uncommon cause of postoperative lower extremity weakness and sensory disturbance [5-7]. Because the sciatic nerve is anatomically distal to the lumbosacral nerve roots, lesions affecting the sciatic nerve may produce motor and sensory deficits that clinically resemble neuraxial complications despite the absence of spinal cord or nerve root injury. Consequently, the diagnosis may be particularly challenging when neurologic deficits develop after spinal anesthesia [5-7]. Electrodiagnostic studies are often required to localize the lesion and differentiate peripheral nerve injury from spinal cord, nerve root, or cauda equina pathology [5-7].

We present a case of unilateral tibial division-dominant sciatic neuropathy that developed after an otherwise uneventful spinal anesthetic in an elderly patient with severe lumbar degenerative disease. This case highlights the diagnostic challenges associated with postoperative neurologic deficits and emphasizes the importance of systematic evaluation, including imaging and electrodiagnostic studies, to distinguish peripheral sciatic neuropathy from neuraxial complications. It also illustrates the potential contribution of preexisting spinal pathology together with perioperative peripheral factors within the framework of the double-crush hypothesis.

## Case presentation

A 70-year-old man (160 cm, 69 kg) with hypertension and hyperlipidemia was scheduled for elective bilateral inguinal hernia repair. His medical history was notable for lumbar spondylosis with L4-5 disc space narrowing and L5-S1 degenerative disease. Several years before surgery, he had experienced symptoms consistent with lumbar spinal stenosis, including neurogenic claudication after walking approximately 50 meters, which improved following conservative treatment.

Premedication consisted of intramuscular midazolam 1 mg and intravenous famotidine 20 mg administered 30 minutes before surgery. Spinal anesthesia was performed in the left lateral decubitus position using a paramedian approach at the L3-4 interspace with a 25-gauge Quincke needle. After confirmation of cerebrospinal fluid flow, 13 mg of 0.5% hyperbaric bupivacaine was administered intrathecally. The patient reported no paresthesia, pain, or discomfort during needle placement or drug injection.

Sensory block reached the T6 dermatome level. The operation lasted approximately 45 minutes and was completed uneventfully. The procedure was performed with the patient in the supine position throughout the operation, and no intraoperative positioning difficulty was documented. Intraoperative hemodynamics remained stable throughout the procedure without episodes of significant hypotension, bradycardia, hypoxemia, or arrhythmia. Estimated blood loss was minimal (approximately 10 mL), and 200 mL of crystalloid solution was administered. The patient experienced mild intraoperative nausea and received ramosetron without further complications. No intraoperative neurologic symptoms or complaints suggestive of nerve irritation were reported.

At approximately two hours after surgery, motor function had recovered in all extremities except the right lower limb. The neurologic deficits persisted throughout the recommended six-hour period of postoperative bed rest, after which the patient continued to experience numbness, paresthesia, and weakness in the right lower extremity, whereas the left lower extremity had recovered completely. On Day 1, he was able to perceive painful stimuli in the right foot but remained unable to flex the toes.

**Figure 1:**
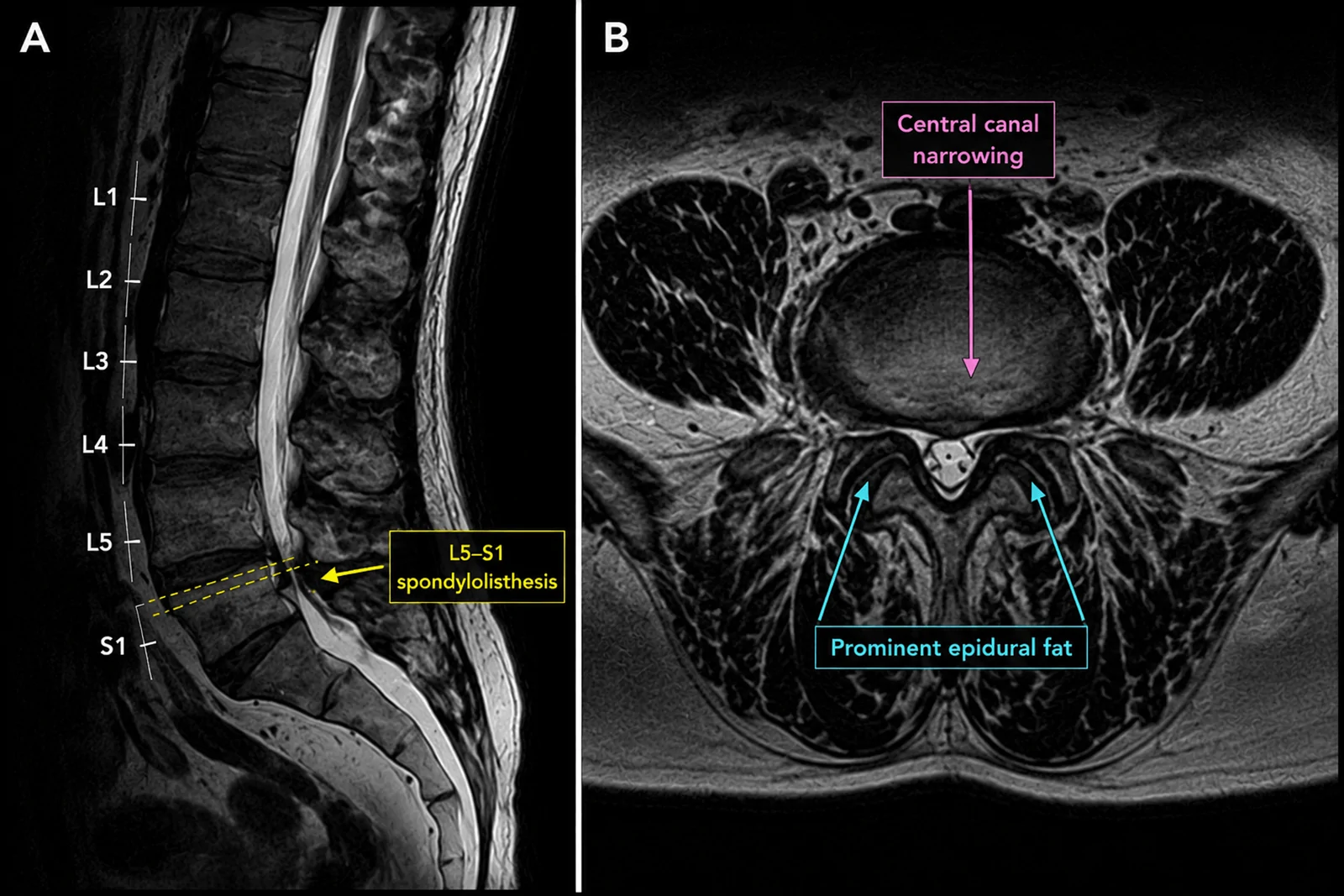
Lumbar magnetic resonance imaging findings. (A) Sagittal T2-weighted image demonstrating marked degenerative lumbar spondylosis with L5-S1 spondylolisthesis and multilevel degenerative disc degeneration. (B) Axial T2-weighted image demonstrating central canal narrowing associated with degenerative changes and prominent epidural fat. No epidural hematoma, spinal cord compression, or other acute neuraxial compressive lesion was identified on non-contrast MRI.

Noncontrast 1.5-T lumbar magnetic resonance imaging, including sagittal T1-weighted, T2-weighted, fat-suppressed T2-weighted, axial T1- and T2-weighted, coronal (oblique) T2-weighted sequences, and a whole-spine survey, was performed on Day 1. The examination demonstrated severe degenerative lumbar disease, including prominent L5-S1 spondylolisthesis, epidural fat hypertrophy, central canal narrowing, and facet arthropathy. No epidural hematoma, spinal cord compression, or other acute neuraxial compressive lesion was identified (Figure 1). 

Neurologic symptoms gradually improved over subsequent days. By Day 3, sensation had partially returned below the knee, and ankle dorsiflexion had improved, although plantar flexion remained impaired. Persistent numbness was noted on the plantar surface of the foot and the lateral aspect of the fifth toe. A consultation with the Department of Physical Medicine and Rehabilitation was performed on Day 3. Mild distal weakness of the right lower extremity and hypoesthesia predominantly involving the S1 dermatome were documented. The patient was able to ambulate with minimal assistance or supervision.

**Figure 2:**
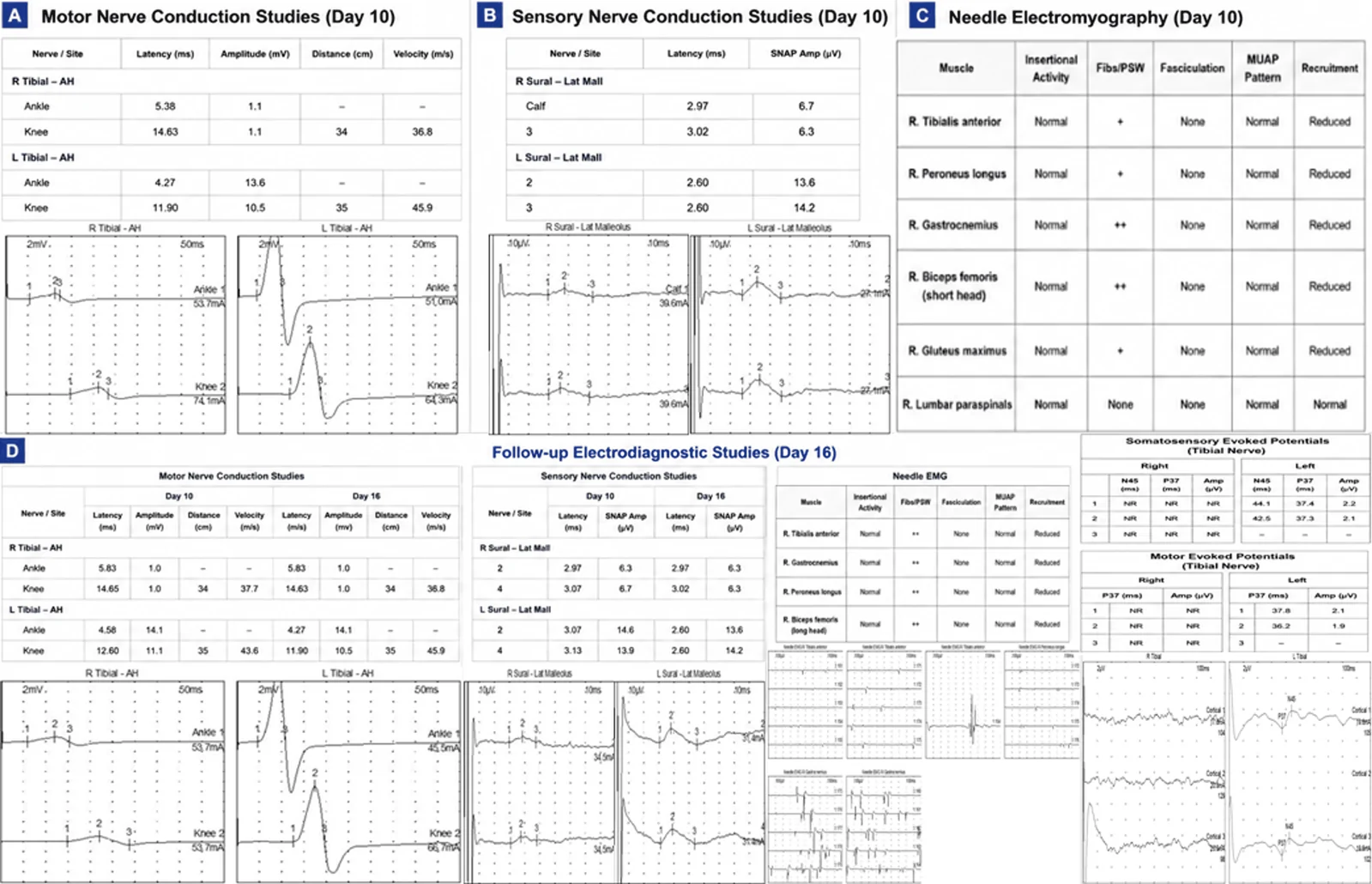
Electrodiagnostic studies demonstrating tibial division-dominant sciatic neuropathy. (A) Motor nerve conduction studies performed on Day 10 demonstrated reduced compound muscle action potential (CMAP) amplitude and slowed conduction velocity in the right tibial nerve. Representative motor nerve conduction waveforms are shown. (B) Sensory nerve conduction studies performed on Day 10 demonstrated reduced amplitude of the right sural nerve sensory nerve action potential (SNAP). Representative sensory nerve conduction waveforms are shown. (C) Needle electromyography performed on Day 10 demonstrated denervation potentials and reduced recruitment in multiple muscles innervated by the sciatic nerve. (D) Follow-up electrodiagnostic studies performed on Day 16 demonstrated persistent tibial division-dominant sciatic neuropathy. Representative motor and sensory nerve conduction studies, needle electromyography, and tibial somatosensory evoked potential (SEP) and motor evoked potential (MEP) findings are shown. The absent right tibial SEP and MEP responses are presented as supportive electrophysiologic findings and were interpreted in conjunction with the nerve conduction study and needle electromyography findings. This figure was prepared by the authors using the original clinical images and electrophysiologic recordings.

Electrodiagnostic studies performed on Days 10 and 16 demonstrated reduced compound muscle action potential amplitudes and conduction velocity in the right tibial nerve, reduced sensory nerve action potential amplitudes in the right sural nerve, denervation potentials in multiple sciatic-innervated muscles, absent tibial somatosensory evoked potentials, and absent tibial motor evoked potentials. These findings were interpreted as consistent with right sciatic neuropathy with predominant involvement of the tibial division. Representative quantitative electrodiagnostic findings are summarized in Table 1, and the corresponding waveforms are shown in Figure 2. These electrodiagnostic findings, particularly the nerve conduction study and needle electromyography results, were concordant with the patient's clinical presentation and supported localization of the lesion to the sciatic nerve rather than the neuraxis. 

**Table 1:** Summary of representative electrodiagnostic findings. CMAP: compound muscle action potential; SNAP: sensory nerve action potential; NCS: nerve conduction study; SEP: somatosensory evoked potential; MEP: motor evoked potential.

Study	Parameter	Right (Affected)	Left (Unaffected)	Interpretation
Motor NCS (Day 10)	Tibial distal latency (ms)	5.38	4.27	Mild prolongation on the right
	Tibial CMAP amplitude (mV)	1.1	13.6	Markedly reduced on the right
	Tibial conduction velocity (m/s)	36.8	45.9	Slower on the right
Sensory NCS (Day 10)	Sural SNAP peak amplitude (µV)	6.7	13.6	Reduced on the right
Motor NCS (Day 16)	Tibial distal latency (ms)	5.83	4.58	Persistent mild prolongation
	Tibial CMAP amplitude (mV)	1.0	14.1	Persistent marked reduction
	Tibial conduction velocity (m/s)	37.7	43.6	Persistent slowing
Sensory NCS (Day 16)	Sural SNAP peak amplitude (µV)	6.3	14.0	Persistent reduction
Somatosensory evoked potentials (Day 16)	Tibial SEP	No response	Present	Absent cortical response on the affected side
Motor evoked potentials (Day 16)	Tibial MEP	No response	Present	Absent motor response on the affected side

**Figure 3:**
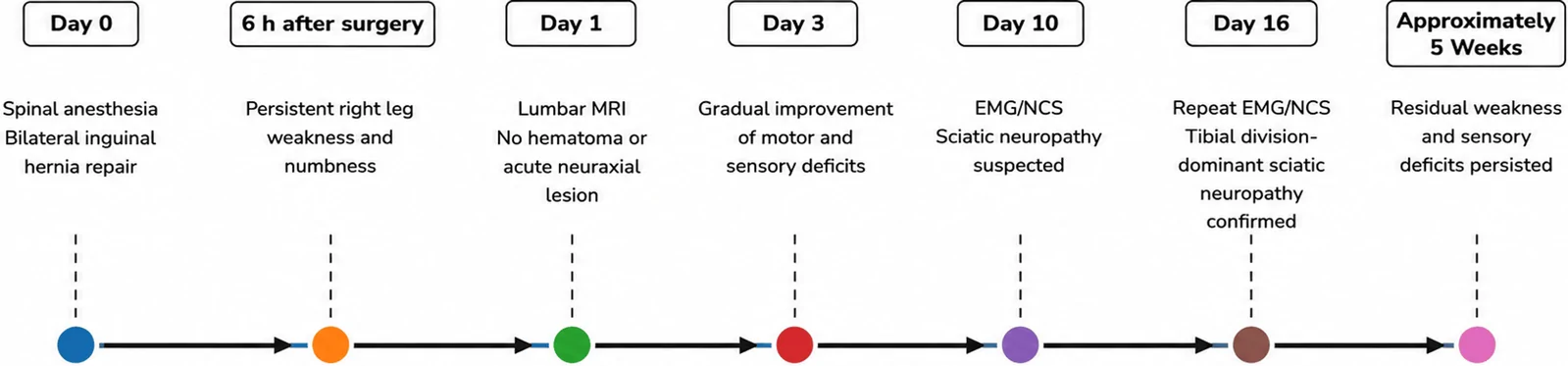
Clinical timeline of postoperative neurologic deficits and diagnostic evaluation. Timeline illustrating symptom onset, neurologic progression, magnetic resonance imaging evaluation, electrodiagnostic studies, and follow-up after spinal anesthesia. Persistent unilateral lower extremity weakness and sensory deficits became apparent after regression of spinal blockade, followed by gradual clinical improvement. Electrodiagnostic studies subsequently demonstrated findings consistent with tibial division-dominant sciatic neuropathy. This figure was prepared by the authors using the original clinical images and electrophysiologic recordings.

Although motor and sensory deficits gradually improved over time, residual weakness and sensory abnormalities persisted at the final outpatient follow-up approximately five weeks after surgery. No additional clinical follow-up data were available thereafter because the patient was lost to follow-up. The chronological clinical course, neurologic findings, and diagnostic evaluations are summarized in Table 2, and the overall clinical timeline is illustrated in Figure 3. 

**Table 2:** Chronological clinical course, neurologic findings, and diagnostic evaluation. PM&R, Physical Medicine and Rehabilitation; MRI, magnetic resonance imaging; EMG, electromyography; NCS, nerve conduction study.

Time Point (Post-op)	Clinical Findings
Day 0 (6 hours after surgery)	Complete recovery of the left lower extremity. Persistent numbness, paresthesia, and weakness of the right lower extremity. Unable to stand without assistance.
Day 1	Partial recovery of pain sensation in the right foot. Toe flexion remained absent. Lumbar MRI demonstrated severe degenerative lumbar disease with L5–S1 spondylolisthesis, epidural fat hypertrophy, and central canal narrowing. No epidural hematoma or acute neuraxial lesion was identified.
Day 3	Gradual improvement in sensory and motor function. Sensation below the knee improved. Dorsiflexion partially recovered, whereas plantar flexion remained impaired. Persistent numbness of the plantar surface and lateral aspect of the fifth toe.
Day 3 (PM&R consultation)	Right distal lower extremity weakness was mild. Hypoesthesia predominantly involved the S1 dermatome. Ambulation was possible with minimal assistance or supervision.
Day 10	Persistent but improving weakness and sensory deficits. EMG/NCS demonstrated reduced CMAP amplitude in the right tibial nerve, reduced SNAP amplitude in the right sural nerve, and incomplete interference patterns in multiple sciatic-innervated muscles. Findings were suggestive of sciatic neuropathy.
Day 16	Follow-up EMG/NCS demonstrated denervation potentials in the tibialis anterior, gastrocnemius, peroneus longus, and biceps femoris muscles. Tibial SEP and MEP responses were absent. Findings were consistent with tibial division-dominant sciatic neuropathy.
Approximately 5 weeks	Residual weakness and sensory abnormalities persisted despite gradual clinical improvement. Long-term neurologic outcome was unavailable because the patient was subsequently lost to follow-up.

## Discussion

Neurologic complications following spinal anesthesia are uncommon but remain among the most feared adverse events associated with neuraxial anesthesia. The differential diagnosis of delayed neurologic recovery following spinal anesthesia includes direct needle trauma, epidural hematoma, epidural abscess, cauda equina syndrome, local anesthetic neurotoxicity, ischemic injury, exacerbation of preexisting neurologic disease, and unrelated peripheral nerve injury [1-4].

In the present case, unilateral sensory and motor deficits developed in the right lower extremity following an otherwise uneventful spinal anesthetic. Because the symptoms became evident after regression of the spinal block and persisted beyond the expected duration of intrathecal bupivacaine, a neurologic complication was initially suspected. Prompt evaluation was therefore necessary to exclude potentially reversible neuraxial pathologies requiring urgent intervention.

One of the most important considerations was epidural hematoma. Although spinal epidural hematoma is rare, delayed diagnosis may result in irreversible neurologic injury. In our patient, noncontrast lumbar MRI performed shortly after symptom onset demonstrated no evidence of epidural hematoma, spinal cord compression, or other acute neuraxial compressive lesions. Although contrast-enhanced imaging was not performed, there were no imaging findings or clinical features suggestive of an epidural abscess. Furthermore, the patient did not exhibit progressive neurologic deterioration, severe back pain, bladder dysfunction, bowel dysfunction, or saddle anesthesia. These findings made epidural hematoma and cauda equina syndrome unlikely explanations for the clinical presentation [1,4].

Direct needle-related neural injury was also considered. During spinal anesthesia, the patient reported no paresthesia, electric shock-like sensation, pain, or discomfort during needle advancement or intrathecal injection. The block was performed without technical difficulty at the L3-4 interspace using a 25-gauge Quincke needle, and no traumatic puncture was documented. Although the absence of paresthesia does not completely exclude needle trauma, the clinical presentation and subsequent electrodiagnostic findings were not characteristic of direct neuraxial injury [3].

Local anesthetic neurotoxicity was similarly considered unlikely. Hyperbaric bupivacaine 13 mg is a standard dose for lower abdominal surgery, and bilateral sensory and motor block regression occurred as expected, except for persistent unilateral deficits. Moreover, neurotoxicity from intrathecal local anesthetics typically presents as neuraxial dysfunction rather than an isolated peripheral neuropathy localized to the sciatic nerve distribution [3,4].

Although spinal anesthesia was performed in the left lateral decubitus position using hyperbaric bupivacaine, the postoperative neurologic deficit developed in the right (nondependent) lower extremity. This laterality is not readily explained by the preferential dependent-side spread of hyperbaric local anesthetic and therefore makes asymmetric intrathecal anesthetic distribution a less likely explanation for the unilateral neurologic deficit.

The temporal association between spinal anesthesia and symptom onset raises concern for an anesthesia-related neurologic complication. However, the absence of paresthesia during needle placement, the lack of radiologic evidence of neuraxial injury, and electrodiagnostic findings localizing the lesion predominantly to the sciatic nerve make a direct neuraxial injury less likely.

Electrodiagnostic evaluation proved crucial in localizing the lesion. Initial nerve conduction studies demonstrated reduced compound muscle action potential amplitudes in the right tibial nerve and reduced sensory nerve action potential amplitudes in the right sural nerve. Follow-up studies revealed denervation potentials in the tibialis anterior, gastrocnemius, peroneus longus, and biceps femoris muscles. In addition, tibial somatosensory evoked potentials and motor evoked potentials were absent. Collectively, the nerve conduction study and needle electromyography findings were interpreted as consistent with sciatic neuropathy with predominant involvement of the tibial division. The electrodiagnostic pattern primarily supported localization of the lesion to the peripheral nervous system rather than the spinal cord or diffuse neuraxial injury. The absent tibial somatosensory evoked potential (SEP) and motor evoked potential (MEP) responses were considered supportive findings but were not used in isolation for lesion localization [5-7].

Another important differential diagnosis is perioperative positioning-related sciatic neuropathy resulting from prolonged compression or stretch of the sciatic nerve. Although peripheral nerve injuries related to positioning should always be considered in patients presenting with postoperative neurologic deficits, this explanation was considered less likely in the present case because the procedure was performed in the supine position, lasted approximately 45 minutes, and no intraoperative positioning difficulties were documented. Furthermore, the electrodiagnostic findings were more consistent with tibial division-dominant sciatic neuropathy than with those of a typical positioning-related compression injury.

Sciatic neuropathy is an uncommon but important cause of postoperative lower extremity weakness and sensory disturbance. The differential diagnosis includes lumbosacral radiculopathy, plexopathy, and central nervous system lesions. Electrodiagnostic studies are often essential for localization because clinical findings may overlap considerably. In the present case, reduced tibial CMAP amplitudes, sural sensory abnormalities, and denervation changes in multiple sciatic-innervated muscles strongly supported the diagnosis of sciatic neuropathy. The absent tibial SEP and MEP responses provided additional supportive evidence but were interpreted in conjunction with the nerve conduction study and needle electromyography findings rather than as localization studies alone. Although the sensory deficit was distributed predominantly in the S1 dermatome on clinical examination, the overall electrodiagnostic pattern favored sciatic neuropathy over isolated S1 radiculopathy [5-7]. The predominant involvement of the tibial division was another notable feature of this case. Although the precise mechanism underlying this selective involvement remains uncertain, previous electrodiagnostic studies have shown that sciatic neuropathy may preferentially involve either the tibial or peroneal division, depending on the underlying site and mechanism of injury [5-7].

An alternative explanation is that preexisting severe lumbar degenerative disease, including central canal narrowing, epidural lipomatosis, and L5-S1 spondylolisthesis, may have rendered the lumbosacral neural structures more vulnerable to perioperative stress. In this context, spinal anesthesia and other perioperative factors may have acted as contributing rather than causative factors, consistent with a "double-crush" mechanism.

The concept of double-crush syndrome, first proposed by Upton and McComas, suggests that neural structures compromised at one site become more susceptible to injury from a second insult occurring elsewhere along the neural pathway [8]. Our patient had a history of symptomatic lumbar spinal stenosis severe enough to cause neurogenic claudication and MRI findings demonstrating marked degenerative changes, central canal narrowing, epidural fat hypertrophy, and spondylolisthesis. Such preexisting pathology may have reduced neural reserve and increased susceptibility to perioperative neurologic dysfunction. In the present case, the proximal site of potential vulnerability was the lumbosacral nerve root region affected by severe degenerative spinal disease, whereas the distal lesion localized electrophysiologically to the sciatic nerve. Thus, the proposed mechanism is consistent with the classic double-crush concept, in which sequential insults occur along the same neural pathway rather than representing two unrelated neurologic disorders [8].

Within the framework of the double-crush hypothesis, the precise nature of the second insult cannot be determined with certainty in the present case. Plausible perioperative contributors include transient mechanical stretch or compression of the sciatic nerve during routine patient positioning, as well as physiologic stress associated with neuraxial anesthesia and surgery. However, the patient remained in the supine position throughout a relatively short procedure, and no intraoperative positioning difficulties were documented, making a major nerve injury related to positioning less likely. Therefore, rather than attributing the neuropathy to a single identifiable event, it is more likely that severe preexisting lumbar degenerative disease reduced neural reserve, allowing an otherwise minor perioperative insult to result in clinically significant sciatic neuropathy.

The proposed vulnerable-patient framework is supported by previous studies examining neuraxial anesthesia in patients with spinal canal pathology. Hebl et al. reviewed 937 patients with spinal stenosis, lumbar radiculopathy, or previous spinal surgery who underwent neuraxial blockade and reported a neurologic complication rate of 1.1%, which was higher than that reported in the general population receiving neuraxial anesthesia [1]. Importantly, the authors concluded that although an increased risk was observed, the relative contributions of the anesthetic technique, surgical procedure, and natural progression of the underlying spinal disease could not be determined. Similar concerns have been raised in subsequent reviews, which identified spinal stenosis and compressive radiculopathy as potential risk factors for postoperative neurologic complications after neuraxial blockade [2,3]. Importantly, these studies do not establish peripheral sciatic neuropathy as a direct complication of neuraxial anesthesia. Rather, they support the concept that patients with preexisting spinal pathology may be more susceptible to postoperative neurologic complications, consistent with the vulnerable-patient framework proposed in the present case [1-3].

Another notable feature of this case was the gradual and spontaneous neurologic improvement observed during hospitalization. Sensory deficits and motor weakness improved progressively over serial examinations, and the patient regained ambulatory function. This recovery pattern is less typical of severe structural neuraxial injury and may instead reflect a partially reversible peripheral nerve injury or decompensation of preexisting neurologic disease. Nevertheless, residual weakness and sensory abnormalities remained at the final follow-up approximately five weeks after surgery, indicating that clinically significant neurologic dysfunction had occurred.

This case highlights several important clinical implications. First, asymmetric delayed recovery after spinal anesthesia should not automatically be attributed to a prolonged local anesthetic effect. Second, urgent imaging is essential to exclude compressive neuraxial lesions. Third, electrodiagnostic studies may provide valuable localization when the diagnosis remains uncertain. Finally, patients with significant lumbar spinal stenosis, spondylolisthesis, or other degenerative spinal disorders may represent a population at increased risk for postoperative neurologic symptoms and therefore warrant careful preoperative counseling and postoperative neurologic assessment. Careful attention to intraoperative positioning and documentation of positioning-related factors may also facilitate the evaluation of postoperative neurologic deficits should they occur [1-3].

This report has several limitations. Because this is a single case report, a definitive causal relationship between spinal anesthesia and the subsequent neurologic deficit cannot be established. In addition, long-term neurologic outcome data were unavailable because the patient was lost to follow-up after the early recovery period. Therefore, the precise mechanism and ultimate prognosis remain uncertain. Nevertheless, the detailed clinical course, serial neurologic examinations, MRI findings, and electrodiagnostic studies provide valuable insight into the evaluation of postoperative neurologic deficits occurring after spinal anesthesia in patients with significant preexisting spinal pathology.

## Conclusions

This case describes unilateral tibial division-dominant sciatic neuropathy that became apparent following an otherwise uneventful spinal anesthetic in a patient with severe preexisting lumbar degenerative disease. Although the temporal relationship between spinal anesthesia and symptom onset initially raised concern for an anesthesia-related neurologic complication, radiologic evaluation revealed no acute neuraxial pathology, and electrodiagnostic studies ultimately localized the lesion to the sciatic nerve.

The present case highlights the diagnostic challenges associated with delayed or asymmetric neurologic recovery after spinal anesthesia. When postoperative neurologic deficits persist beyond the expected duration of spinal blockade, prompt evaluation is essential to exclude potentially reversible neuraxial complications. In addition to neuroimaging, electrodiagnostic studies may provide valuable localization and assist in differentiating peripheral neuropathy from neuraxial injury. Furthermore, patients with significant preexisting spinal pathology may be more vulnerable to postoperative neurologic dysfunction, emphasizing the importance of careful neurologic assessment, appropriate counseling, and close postoperative follow-up. Although a definitive causal relationship could not be established, this case underscores the importance of a systematic diagnostic approach and careful neurologic follow-up when neurologic deficits occur after neuraxial anesthesia.
